# Viral Encoded miRNAs in Tumorigenesis: Theranostic Opportunities in Precision Oncology

**DOI:** 10.3390/microorganisms10071448

**Published:** 2022-07-18

**Authors:** Rodney Hull, Rahaba Marima, Mohammed Alaouna, Demetra Demetriou, Rui Manuel Reis, Thulo Molefi, Zodwa Dlamini

**Affiliations:** 1 SAMRC Precision Oncology Research Unit (PORU), DSI/NRF SARChI Chair in Precision Oncology and Cancer Prevention (POCP), Pan African Cancer Research Institute (PACRI), University of Pretoria, Hatfield 0028, South Africa; rodneyhull@gmail.com (R.H.); rahaba.marima@up.ac.za (R.M.); 321814@students.wits.ac.za (M.A.); demetrioudemetra223@gmail.com (D.D.); ruireis.hcb@gmail.com (R.M.R.); 2 Department of Internal Medicine, Faculty of Health Sciences, School of Clinical Medicine, University of the Witwatersrand, Parktown 2193, South Africa; 3 Life and Health Sciences Research Institute (ICVS), School of Medicine, University of Minho, 4710-057 Braga, Portugal; 4 Molecular Oncology Research Center, Barretos Cancer Hospital, Antenor Duarte Villela, Barretos 1331, São Paulo 14784-400, Brazil; 5 Department of Medical Oncology, Steve Biko Academic Hospital, University of Pretoria, Hatfield 0028, South Africa

**Keywords:** viral-miRNAs (v-miRNAs), human papillomaviruses (HPV), Hepatitis C virus (HCV), Epstein–Barr virus (EBV), Hepatitis B virus (HBV), Merkel Cell Polyomavirus (MCPyV), Kaposi’s sarcoma-associated herpesvirus (KSHV), biomarkers, therapeutics, theranostics, precision oncology

## Abstract

About 15% of all human cancers have a viral etiology. Although progress has been made, understanding the viral oncogenesis and associated molecular mechanisms remain complex. The discovery of cellular miRNAs has led to major breakthroughs. Interestingly, viruses have also been discovered to encode their own miRNAs. These viral, small, non-coding miRNAs are also known as viral-miRNAs (v-miRNAs). Although the function of v-miRNAs largely remains to be elucidated, their role in tumorigenesis cannot be ignored. V-miRNAs have also been shown to exploit the cellular machinery to benefit viral replication and survival. Although the discovery of Hepatitis C virus (HCV), and its viral miRNAs, is a work in progress, the existence of HPV-, EBV-, HBV-, MCPyV- and KSHV-encoded miRNA has been documented. V-miRNAs have been shown to target host factors to advance tumorigenesis, evade and suppress the immune system, and deregulate both the cell cycle and the apoptotic machinery. Although the exact mechanisms of v-miRNAs-induced tumorigenesis are still unclear, v-miRNAs are active role-players in tumorigenesis, viral latency and cell transformation. Furthermore, v-miRNAs can function as posttranscriptional gene regulators of both viral and host genes. Thus, it has been proposed that v-miRNAs may serve as diagnostic biomarkers and therapeutic targets for cancers with a viral etiology. Although significant challenges exist in their clinical application, emerging reports demonstrate their potent role in precision medicine. This review will focus on the roles of HPV-, HCV-, EBV-, HBV-, MCPyV-, and KSHV-produced v-miRNAs in tumorigenesis, as effectors in immune evasion, as diagnostic biomarkers and as novel anti-cancer therapeutic targets. Finally, it will discuss the challenges and opportunities associated with v-miRNAs theranostics in precision oncology.

## 1. Introduction

Identifying cellular miRNAs has been essential in understanding cancer biology in recent decades. Viral-miRNAs (v-miRNAs) were identified soon after cellular miRNAs. Although cellular and v-miRNAs have comparable biogenesis pathways, the involvement of v-miRNAs in tumorigenesis remains largely unknown. DNA, RNA, and retroviruses can be found to encode v-miRNAs. According to some studies, oncovirus-associated cancers are more prevalent in immunocompromised people [1]. It has also been reported that tumor-suppressor signaling and innate immune signaling share similar effector proteins and/or pathways, such as p21 and p53. v-miRNAs also play an essential role in viral proliferation and persistence. It has been shown that v-miRNAs and cellular miRNAs can influence both host and virus-derived transcripts [2]. This may be possible if both v-miRNA and cellular miRNA share similar seed sequences (miRNA sequence complementary to the mRNA target, 2-8nt from 5′ to 3′ end) and targeted regulation. The relationship between v-miRNA and cellular miRNA orthologues largely remains to be elucidated. It has also been shown that kshv-mir-K12-11 is similar to hsa-miR-155 [3]. The DNA sequences of kshv-mir-K12-10 and hsa-mir-142-3p have a significant degree of sequence similarity [4]. It has also been shown that the seed sequences of both ebv-miR-BART-5 and hsa-miR-18 are similar [4]. Due to the multifaceted nature employed by the v-miRNAs, these molecules may play an important role in precision oncology, particularly in cancers with a viral etiology. Precision oncology can be defined as the molecular profiling of tumors to identify targetable alterations [5]. Cellular miRNAs have also been identified as potent alterations that can be profiled for use in precision oncology [6].

**Figure 1:**
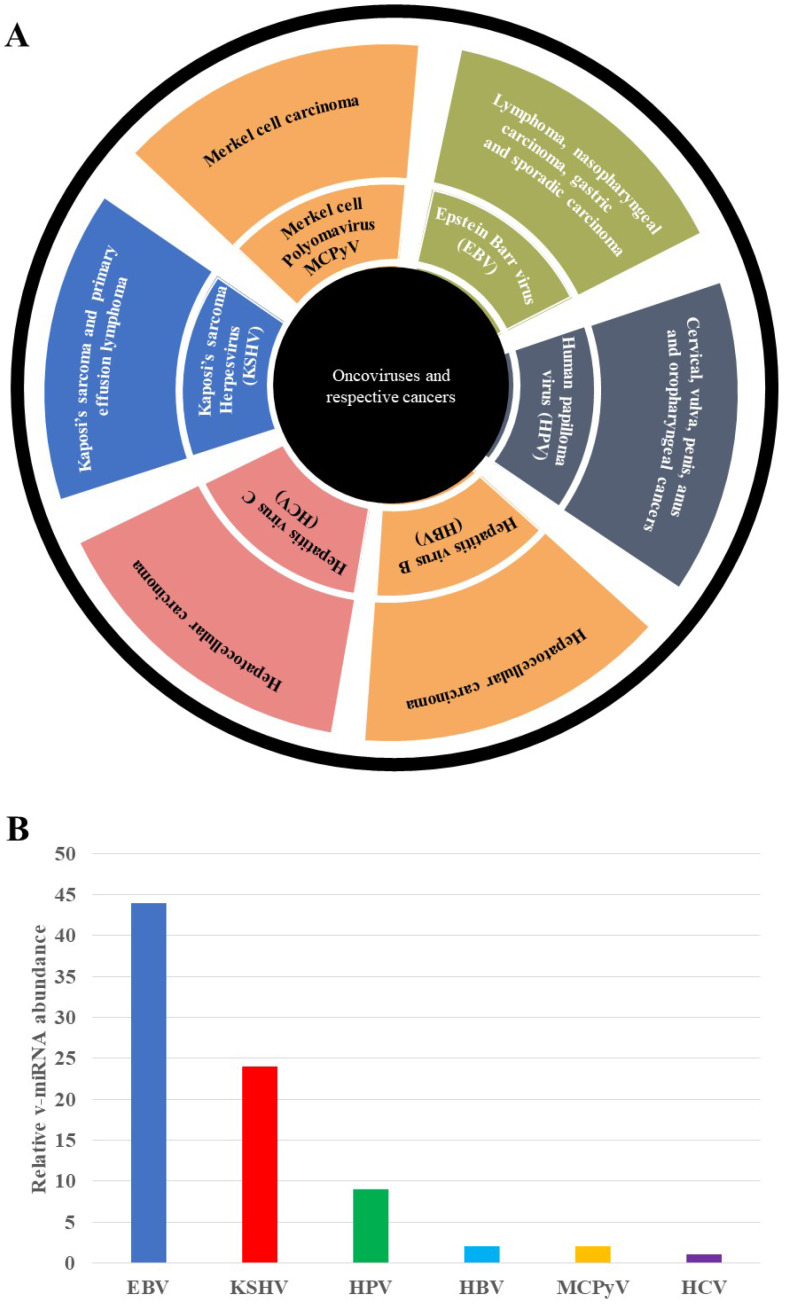
**Oncogenic viruses in precision oncology.** (**A**) Summary of oncoviruses and their associated tumors. These oncoviruses include EBV, HPV, KSHV, HBV, HCB and MCPyV. Cancers associated with these viruses include Hodgkin’s lymphoma, cervical cancer, Kaposi sarcoma, liver cancer and Merkel cell carcinomas. (**B**) Representation of relative v-miRNA abundance. EBV v-miRNAs relative abundance is higher than the other v-miRNAs, followed by KSHV, HPV, HBV, and no HCV* v-miRNA has been confirmed to date [10].

Cellular factors are exclusively involved in v-miRNA biogenesis and the mature v-mRNAs are exported through exosomes [2,7]. V-miRNAs have immunomodulatory effects, influencing the host’s innate and adaptive immune systems [7]. These v-miRNAs’ immunomodulatory mechanisms confer viruses with the ability to escape the host’s immunosurveillance. Furthermore, v-miRNAs permit viruses to enter their latent phases. These viruses can thus evade detection by immune surveillance systems, increasing the probability of cancer formation [8]. Chronic infection has been shown to upregulate cell adhesion and migration, the cell cycle, immune response and blocking regulation of critical physiological processes [9]. Viruses have also evolved a complicated symbiotic mechanism for accessing and regulating host transcriptional machinery. This review will discuss the roles of Human Papilloma virus (HPV)-, Hepatitis C virus (HCV)-, Epstein Barr virus (EBV)-, Hepatitis B virus (HBV)-, Merkel cell polyomavirus (MCPyV)-, and Kaposi’s sarcoma associated Herpes virus (KSHV)-encoded v-miRNAs in tumorigenesis, v-miRNAs’ role in immune evasion, v-miRNAs as a diagnostic biomarker and as novel anti-cancer therapeutic targets in precision oncology, and, lastly, the challenges and opportunities associated with v-miRNAs in clinical research. Figure 1A shows oncoviruses and their associated cancers, with EBV and HPV having the highest number of different cancers being associated with infection with these viruses. Figure 1B illustrates the relative abundance of v-miRNAs detected in EBV, KSHV, HPV, HBV, MCPyV and HCV to date. EBV has had the highest number of miRNAs identified in its genome. This is followed by KSHV, with nearly two thirds the number of miRNAs as EBV and HPV, with a significantly lower number: nearly a fifth of the miRNAs identified in EBV. The remaining viruses all have very low numbers of isolated v-miRNAs.

## 2. Viral-miRNA (v-miRNA) Biogenesis and Role in Tumorigenesis

**Figure 2:**
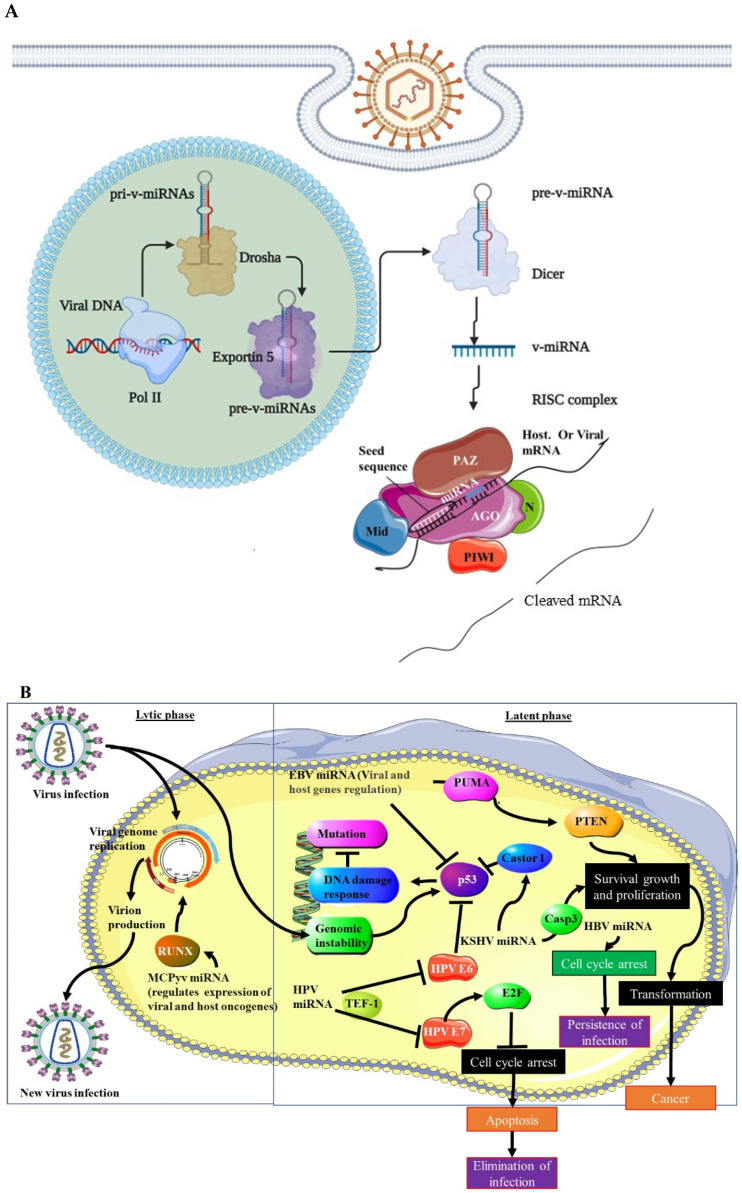
**V-miRNAs’ mediated tumourigenesis.** (**A**) Schematic representation of v-miRNA biogenesis. Simliar to cellular miRNAs, v-miRNAs are transcribed as primary v-miRNAs (pri-v-miRNAs) in the nucleus. Pri-v-miRNAs is then cleaved by Drosha into pre-v-miRNA. Exportin 5 exports the pre-v-miRNA to the cytoplasm. Mature v-miRNA is generated from pre-v-miRNA by Dicer cleavage. MiRNA is then loaded into the RISC complex to target mRNA, viral and cellular transcripts [11]. (**B**) Diagrammatic representation of v-miRNA mediated tumorigenesis. V-miRNAs play an important role in viral latency by targeting key tumour-suppressor genes such as p53 and PTEN, causing genomic instability, and favouring tumorigenic pathways such as cell growth, survival, proliferation and cell transformation, and immune evasion, while inhibiting anti-tumorigenic mechanisms such as cell cycle arrest and apoptosis [10].

Viruses utilise the same methods as their host to generate miRNAs that will encode their v-miRNAs. To generate v-miRNAs, *Dicer* and *Drosha* must be active [11,12,13]. Primary miRNA is created by the transcribed viral miRNA gene (pri-miRNA). The microprocessor complex DiGeorge syndrome critical region 8 (DGCR8) processes v-pri-miRNA in the nucleus to create pre-v-miRNA with a nucleotide length of 70nt. The Exportin-5 protein transports the pre-v-miRNA from the nucleus to the cytoplasm. In the cytoplasm, Dicer endonuclease transforms pre-v-miRNA into a mature v-miRNA duplex (21–25nt in length). This is subsequently loaded into the RNA-induced silencing complex (RISC), which comprises Argonaute 2 (Ago2) and other components. Figure 2A depicts v-miRNA biogenesis. The v-miRNA-RISC complex then suppresses the target transcript [11,14]. This repression follows the binding of the v-miRNA to the complementary sequence on the target mRNA. This guides RISC to this target site with the most complementarity, usually in the 3’untranslated region of the miRNA. The seed region is the region that must, in mmost cases, be fully complementary for RISC to act on the mRNA. In a quarter of cases, there may be some incomplete complementarity between the RNA and the seed sequence. Once RISC has been guided to this site, the mRNA may undergo end nucleolytic cleavage through the Ago2 action [15]. V-miRNAs can be found using computational techniques or by sequencing tiny, cloned RNA molecules. The latter strategy has the potential to be more accurate than the former. The sequencing of small, cloned RNA molecules can limit the detection of less abundant v-miRNAs, which can be detected using computational approaches [16,17]. V-miRNAs have also been reported to regulate the cellular splicing machinery (reviewed in [18]). In addition to this V-miRNAs affect the cellular pathways involved in tumorigenesis. These pathways include cell growth, cell proliferation, cell proliferation and survival, cell death, DNA damage response, and cell cycle arrest. V-miRNAs can also evade the immune system by transitioning from lytic to latent phases, and, due to their micro-size, may escape immune defense (reviewed in [10]). Due to their dual (host and viral) regulatory mechanisms, the network of v-miRNA targets is broad. Figure 2B illustrates v-miRNA-mediated tumorigenic pathways.

## 3. Human Papillomaviruses (HPV)

### 3.1. HPV Oncovirus

HPV is a DNA virus that infects the mucosa and cutaneous epithelial cells [10,19]. The circular DNA HPV genome encodes for six nonstructural genes, which are *E1*, *E2*, *E4*, *E5*, *E6*, and *E7*, and two *L1* and *L2* viral assembly genes. The *E1*, *E2*, and *E4* genes are involved in HPV viral replication, while the remaining three non-structural genes *E5*, *E6*, and *E7* are involved in HPV viral-induced cellular transformation [20,21]. High-risk HPV 16 and 18 infections are responsible for most cervical cancer cases [22,23]. Most HPV infections are cleared through surveillance by the immune system. Recurring and persistent viral infections lead to viral integration, immune evasion and, eventually, to malignancy [22]. The E6 and E7 HPV oncoproteins deregulate key host tumor-suppressors, p53 and retinoblastoma protein (pRb), and promote malignancy. HPV v-encoded miRNAs have been documented [24,25].

### 3.2. HPV v-miRNAs

HPV-encoded v-miRNAs have also been documented. Chirayil et al. discovered that HPV 17, 37, and 41 are involved in the generation of HPV-miRNA [17]. Weng et al. discovered that the number of HPV-encoded miRNAs differs between HPV species [8]. In cervical tissue samples, Virtanen et al. identified the HPV-16-miRNAs miR-H1, miR-H3, miR-H5, and miR-H6 [26]. HPV-16-encoded miRNAs have also been demonstrated to target transcription enhancer factor 1 (TEF-1) [9]. TEF-1 is a transcription factor that affects cell proliferation and migration. TEF-1 also binds to and activates the *E6* and *E7*, which are early promoters of HPV 16 [27,28]. Excessive cell proliferation is partly caused by E6 attaching to and degrading p53 and partly by E7 degrading retinoblastoma (RB) and interrupting the cell cycle [29,30]. HPV-miRNAs seem to indirectly regulate tumorigenesis s by regulating the HPV E6 and E7 oncogenic proteins through TEF-1. This process promotes cell-survival mechanisms that ultimately lead to tumorigenesis. Similarly, HPV-miRNAs regulate proteins key in immunosurveillance mechanisms [31]. Although significant progress has been achieved in the functional identification of HPV-miRNAs, further research is needed to identify new HPV-miRNAs and determine their specific involvement in tumorigenesis. HPVs can cause cervical, penile, anal, and head and neck cancers. HPV-miRNA profiling has been carried out to uncover new biomarkers for diagnosis, prognosis, and improved therapy [32,33]. It is important to specify the viral status of HPV-associated and non-HPV-associated cancers, as the two groups are etiologically distinct. Table 1 shows a summary of v-mRNAs with oncogenic roles, and their targets and cellular effects.

## 4. Hepatitis B Virus (HBV)

### 4.1. HBV Oncovirus

The hepatitis viruses (B and C) have been reported as indirect carcinogens, compared to other viruses such as HPV [10,80]. HBV is a hepatotropic virus belonging to the Hepadnaviruses family. It possesses a partly dsDNA genome, which is converted to a covalently closed circular DNA by the cellular machinery [81]. Although the exact molecular mechanisms remain to be elucidated, HBV infection is implicated in over 50% of hepatocellular carcinomas. Four viral genes are encoded by the HBV genome: *HBcAg*, *HBx*, *DNA polymerase* and *HBsAg* [22,82]. HBV v-miRNAs have been reported in tumorigenesis (reviewed in [83]).

### 4.2. HBV v-miRNAs

In hepatocellular carcinoma (HCC), a newly identified HBV-miRNA (HBV-miR-2) serves as a tumor-suppressor and an oncogenic miRNA by inhibiting Tripartite Motif Containing 35 (TRIM35) and targeting Ras-related nuclear protein (RAN). TRIM35 slows cell growth and triggers apoptosis, both of which are hallmarks of its tumor-suppressing abilities. RAN overexpression, on the other hand, increases the transformation of epithelial to mesenchymal cells (EMT) into HCC cells [84]. Ovarian cancer and HCC proliferative pathways have been related to RAN [33]. Furthermore, an indirect mechanism by which HBV-miRNAs can induce tumorigenesis has been proposed. HBV-miR-3, for example, suppresses HBV protein production by targeting the mRNAs for HBVsAg, HBeAg, and Hc and interrupts HBV’s intermediate DNA replication [85]. HBV miR-3 targets HBV 3.5 kb transcript, reducing HBc and pre-genomic RNA levels, resulting in decreased HBV replication. Long-term infection, as well as repeated HBV-induced liver inflammation, all contribute to the development of hepatocellular carcinoma (HCC) [85].

Non-lymphoma B-cell Hodgkin’s disease (B-NHL) [86] and nasopharyngeal cancer have been linked to HBV infection [87]. In the HBV genome, there are four open reading frames (ORFs). Surface protein genes (*preS1*, *preS2*, and *S*) are included in these ORFs, as are core protein genes, polymerase genes, and genes that encode the HBx component [88]. When compared to other DNA viruses, research on HBV-encoded miRNAs is still in its infancy in terms of the identity, transcription and function of HBV v-miRNAs. Table 1 provides a summary of various v-miRNAs, including HBV v-miRNAs.

## 5. Merkel Cell Polyomavirus (MCPyV)

### 5.1. MCPyV Oncovirus

Merkel cell polyomavirus (MCPyV) is a dsDNA mammalian polyomavirus [89]. This virus causes lifelong, persistent infection in host cells [90]. The ~5 kbp-long MCPyV genome encodes for 2 early long and small antigens, and 2 late structural antigens [10,91,92]. Persistent MCPyV infection in immunocompromised patients has been reported to cause Merkel cell carcinoma [93,94].

### 5.2. MCPyV v-miRNAs

The miRNAs mcv-miR-M1-5p and mcv-M1-3p of Merkel Cell Polyomavirus (MCPyV) have been identified. Both v-miRNAs were made from a single miRNA precursor [10,95,96,97]. Mcv-miR-M1 miRNAs have been demonstrated to influence viral life cycle transitions from early to late stages by modulating viral gene expression and they may also play a role in immunosurveillance evasion [95,98]. It was also found that sequence of miR-M1-seed 5p’s was linked to the *sp100i* gene which codes for the nuclear antigen SP100. This resulted in the downregulation of interleukin 8, which, in turn, led to a decrease in the host immunological response [97]. Moreover, an expanded list of the mcv-miR-M1 miRNA human gene targets has been reported [98]. These gene targets include *phosphatidylinositol-4,5-bisphosphate 3-kinase catalytic subunit delta (pik3cd)*, *proteosome activator subunit 3(psme3)* and *runx1* [99,100,101,102]. PIK3CD and PSME3 are responsible for host cell antigen presentation, while RUNX1 is a transcription factor that can either promote or inhibit tumorigenesis [98,103,104,105].

MCpyV is detected in over 90% of Merkel cell carcinomas (MCCs) [92]. Early in viral oncogenesis, small T (ST) and large T (LT) antigens are encoded [32,106]. MCPyV also produces two early-stage proteins, ALTO and 57kT, which are encoded by the alternative LT open reading frame. VP1, VP2, and VP3 are all proteins expressed at the later stages of infection [107]. A summary of MCPyV v-miRNAs, including their targets and effects, is shown in Table 1.

## 6. Epstein–Barr Virus (EBV)

### 6.1. EBV Oncovirus

EBV was the first oncovirus of the herpesvirus family to be identified. EBV associated cancers include Burkitt’s lymphoma, Hodgkin’s lymphoma, B and T cell lymphomas and gastric cancer [22,108,109]. EBV is a lymphotropic herpesvirus and can infect >95% of persons during the early stages of life, and usually causes asymptomatic infection. EBV resides in the memory B cells of infected persons following primary infection [10,110,111]. EBV can also target T cells and the natural killer cells during malignancy [22,108]. Furthermore, EBV has latent and lytic phases in its life cycle [10,112]. EBV encodes six nuclear antigens: EBNA1, EBNA2, EBNA3A, EBNA3B, EBNA3C and EBNA-LP. Additionally, it encodes for three latent membrane proteins, LMP1, LMP2A and LMP2B. Finally, it also encodes a BCL-2 viral homologue, BHRF1. EBV viral oncoproteins such as LMP1, EBNA1, 2 and 3C are responsible for the malignant transformation of the B cells [22,108,109,113].

### 6.2. EBV v-miRNAs

V-miRNAs were first discovered in an EBV-infected cell line [114]. Two miRNA gene clusters, BamH1 fragment H rightward (BHRF1) and BamH1 A region transcripts (BART), encode these EBV-miRNAs. Recently, more than 40 EBV-miRNAs have been discovered [115]. One of the advantages of v-miRNAs for oncoviruses is their ability to regulate virus and host gene expression without producing viral proteins. This mechanism allows for infected cells to be invisible to the host immune system, and therefore plays a role in virus latency (reviewed in [32]). Many EBV BART miRNAs (miR-16, -17-5p, and-1-5p) have been shown to modify viral gene transcripts for latent membrane protein 1 (LMP1), leading to EBV-infected cell transformation [116]. Anti-LMP2A miR-BART22 in nasopharyngeal carcinoma facilitates the escape of EBV-infected cells from immune surveillance [44]. Additionally, miR-BART2 downregulates the EBV DNA polymerase, BALF5, inhibiting the lytic phase of viral genome replication [117]. Furthermore, miR-BART6-5p. This has been reported to suppress the EBNA2 viral oncogene, promoting viral latency. It is important to note that miR-BART6-5p only promotes EBV type I and II latency, rather than type III latency [118]. EBV miRNAs may contribute to cancer development through their regulation of host mRNAs. On the other hand, miR-BART5-5p and miR-BART19-5p have been demonstrated to interfere with the p53-upregulated mediator of apoptosis (PUMA) protein’s ability to control and promote apoptosis [119]. The phosphatase and tensin homolog (PTEN) was also shown to be downregulated by miR-BART7-3p in nasopharyngeal cancer cells, promoting EMT and metastasis [120]. Apart from that, miR-BART1 and 16 target *caspase3* (CASP3), which prevents apoptosis in cells [121]. Additionally, the tumor-suppressor deleted in cancer cells 1 (DICE1), also known as integrator complex subunit 6 (INTS6), was also shown to be downregulated by miR-BART3, which resulted in increased proliferation and transformation in nasopharyngeal cancer cells [122]. MiR-BHRF1-3 was reported to suppress the host-interferon-inducible cytokine *C-X-C motif chemokine 11* (*cxcl11*) mRNA, facilitating immune evasion [89]. The targeted inhibition of CXCL11 may be one of the immunomodulating strategies employed by EBV in tumorigenesis.

The miR-BHRF1 v-miRNA cluster improves EBV’s ability to infect the body and promotes acute infection, enhancing the virus-induced B cell transformation [123]. MiR-BART2-5p specifically targets MHC class 1 polypeptide-related sequence B (MICB) molecules, which appear on the surface of infected cells and detected by NK cells as a sign of infection [124,125]. EBV-miR-BART20-5p has also been linked to a poor prognosis in individuals with EBV-related gastric cancer [126]. Glucosamine—fructose-6-phosphate aminotransferase isomerizing 1 (GFPT1/TGF1) signaling is targeted by miR-BART7, which regulates DNA repair in nasopharyngeal cancer cells and predicts treatment success [127]. Iizasa et al. (2010), showed that miR-BART6-5p acts as a negative feedback loop to affect other EBV miRNA expression, as well as its own expression [118]. MiR-3, a tumor-suppressor that inhibits MCM2 protein, is repressed in nasopharyngeal cancer cells [128]. The expression of cellular miR-155 has also been associated with nasopharyngeal cancer and B-cell immortalization [129]. EBV proteins EBV LMP1 and LMP2A regulate cellular miR-155 expression [130,131]. Furthermore, the miR-183-96-182 cluster was demonstrated to be suppressed by LMP1 protein in BL cell lines, mediating EBV cell transformation [132]. LMP1 was also shown to inhibit miR-1 expression in nasopharyngeal cancer [133]. As a tumor-suppressor, MiR-1 inhibits angiogenesis and tumorigenesis by targeting the expression of the Kirsten rat sarcoma (K-ras) gene. A summary of EBV v-miRNAs, including their targets and physiological effects, is listed in Table 1.

## 7. Kaposi’s Sarcoma-Associated Herpesvirus (KSHV)

### 7.1. KSHV Oncovirus

KSHV is also known as the human herpesvirus 8 (HHV-8). KSHV is a linear dsDNA oncovirus belonging to the Herpesviridae family [10]. Similar to EBV, KSHV possesses the ability to establish a chronic infection in lymphocytes, which act as the primary reservoir. Furthermore, KSHV can also form chronic infection in keratinocytes, macrophages, and endothelial cells [134,135,136]. KSHV-induced cellular transformation is associated with immunosuppressed tumors such as Kaposi sarcoma, B cell lymphoproliferative diseases/pleural effusion lymphoma (PEL) and multicentric Castleman disease [137,138]. KSHV encodes for >90 ORFs, with highly conserved genes being found in ORF4-75 [10]. KSHV gene expression patterns determine the lytic and latent switch, with the viral latent genes being transcribed from the constitutively active Latency Associated Nuclear Antigen promoter (LANA) (LTc) and the kaposin promoter located downstream of LANA, LTd (distal latent promoter,) promoters, while the LTi-inducible latent promoter acts as a lytic phase switch [22,139]. KSHV v-miRNAs have also been documented.

### 7.2. KSHV v-miRNA

KSHV encodes a total of 25 miRNAs, which are derived from 12 viral pre-miRNAs. The Kaposin promoter regulates clusters of KSHV miRNA genes during viral latency. Many KSHV pre-miRNA genes appear to be intergenic, positioned between the Kaposin gene sequence and the open reading frame (ORF) 71 of the KSHV genome (reviewed in [72]). KSHV miR-K10, which is situated in the ORF of the Kaposin gene, and miR-K12, which is likewise located in the Kaposin gene, are the only two exceptions to this norm [140]. As in other oncoviruses, KSHV miRNAs, may contribute to cancer development by controlling viral replication and cellular gene expression. In viral oncogenesis, the latent period is critical. MiR-K9-5p and miR-K7-5p, which target the KSHV R trans-activator (RTA) protein, are also targets of the same KSHV R trans-activator (RTA) protein. This protein is in charge of inducing viral lytic activity [134,135]. Other miRNAs that modulate RTA do so by targeting both nuclear factor I/B and G-protein-coupled receptor kinase 2 (GPCRK2) in the cell nucleus [136,141]. In addition, miR-K12-11 reduces RTA activity by targeting the transcription factor MYB for cellular myeloblastosis (which activates the RTA promoter). Furthermore, miR-K12-11 targets IKK epsilon (IKK), which regulates interferon signaling and promotes latency of the KSHV virus [142]. DNA methyl-transferase-1 (DNMT1) also methylates the RTA promoter, maintaining viral latency. V-miR-K12-4-5p controls DNMT1 activity by repressing retinoblastoma-like protein 2 (RBL2) in favor of viral latency [143].

KSHV miRNAs also influence angiogenesis and dissemination in Kaposi sarcoma. MiRNAs such as miR-K12-1, miR-K12-11, and miR-K3-3p inhibit the synthesis of the angiogenesis antagonist Thrombospondin 1 (THBS1). The downregulation of THBS1 causes errors in angiogenesis and uncontrolled cell growth [144]. The STAT3 pathway is stimulated by V-miR-K6-3p, resulting in the migration and invasion of Kaposi sarcoma cells [78]. Cell-cycle regulation by KSHV miRNAs promotes cell survival and cell transformation events. It has been reported that these viral pro-survival mechanisms are promoted by the suppression of aerobic glycolysis and oxidative phosphorylation under nutrient deprivation [145]. V-miR-K12-1 inhibits p21 expression, promoting pro-survival mechanisms in KSHV-infected cells [68]. KSHV-infected cells develop abnormally because the KSHV miRNAs miR-K12-1, miR-K12-3, and miR-K12-4-3p block CASP3 activity [146,147]. The seed sequences of KSHV miRNAs and cellular miRNAs may be similar, as in the case of miR-155 and miR-K12-11. This viral miRNA is able to control both miR-155 and mRNA transcripts that are important in B-cell development by the same KSHV target sequence [75,77]. Table 1 includes a summary of known KSHV v-miRNAs, their targets and their physiological roles.

## 8. Hepatitis C Virus (HCV)

### HCV Oncovirus and v-miRNAs

HCV is an RNA virus and belongs to the family of Flaviviridae [10,22,108,148]. Like HBV, HCV primarily infects hepatocytes and plays a major role in hepatocellular tumorigenesis. The positive strand HCV RNA genome has one ORF that produces various viral proteins. These viral proteins are E1 and 2, NS1, 2, 3, 4A, 4B, 5A and 5B. Following infection, HCV viral RNA is translated using cellular ribosomes. The HCV-positive RNA strand is copied to generate a replicative intermediate with the HCV-negative strand. HCV oncovirus has been reported to induce tumorigenic transformation through double-strand brakes and inducing cellular oxidative stress [10,149,150]. The host oncogenic miRNA, has-miR-122, targets the HCV genome by binding to both the 5′ and 3′UTRs of the viral genome. This results in the upregulation of HCV replication [151]. There are also various studies reporting HCV effects on cellular miRNAs and the effect of cellular miRNAs’ on the HCV life-cycle [152,153,154,155,156,157,158,159,160,161,162]. MiRNA biosynthesis and RNA virus replication are two separate processes, and this separation impedes the splicing process for pri-miRNA generation, as the Drosha enzyme is located in the nucleus [163]. However, evidence of HCV-encoded miRNA is still missing.

## 9. Persistent Viral Infection and Oncogenesis: v-miRNAs as Effectors of Immune Evasion

To establish persistent long-term infection, it is necessary that the virus evade the host innate and adaptive immune responses in the long term, for years or decades. It has been reported that EBV and KSHV viruses are able to establish long-term persistent infection and remain dormant [164,165]. While HPV, HBV, HCV and MCPyV can also establish persistent infection, viral dormancy is not evident. Viral genome integration into the host genome is key to persistent infection, with most of the viral oncoproteins and viral non-coding RNAs, such as v-miRNAs, being produced during viral latency, ensuring the survival of the infected cells and continued presence of the virus [166,167]. Reprogramming of the metabolic profiles in infected cells is also key in the maintenance of viral latency. Additionally, persistent viral infection also induces genomic instability by compromising cellular checkpoints in infected cells, eventually promoting tumorigenesis. Like cellular miRNAs, v-miRNAs can target and regulate viral gene expression (including cellular gene expression) to modulate the active and latent switch modes. Maintenance of the viral latent phase is key to viral immune evasion and v-miRNAs are active participants in this process (reviewed in [168]). For example, EBV BART miRNAs play a significant role in controlling the viral life cycle, and thus maintaining latency. miR-BART16, 17-5p and 1-5p target the LMP1 gene, resulting in excessive cell proliferation [116]. Furthermore, EBV miR-BARTT2 and miR-BARTT20 promote the latent phase by repressing BALF5 DNA pol and suppressing the early EBV genes, BZLF1 and BRLF1 [117,169]. In addition, the KSHV miRNA, miRK9, has also been reported to facilitate KSHV viral latency by suppressing the viral protein RTA, which is responsible for the lytic switch [170]. KSHV miRK12-5 has also been documented to play a role in RTA protein deregulation, favouring latency [70]. Evidence supporting v-miRNA regulation of viral gene expression to promote and maintain viral latency remains to be elucidated and this information would assist in providing key breakthroughs in understanding v-miRNA-mediated immune evasion and tumorigenesis in cancers with a viral aetiology (Reviewed in [115]).

## 10. V-miRNAs’ Diagnostic Significance in Clinical Research and Precision Oncology

The potential use of miRNAs in oncology was first explored when it was demonstrated that the expression profiles s of miRNAs in chronic lymphocytic leukemia were different to those of healthy patients [171]. Subsequently, differential miRNA expression in different cancers has been documented, and this is also been applicable in other non-communicable diseases, such as diabetes and Alzheimer’s disease [172,173,174]. As such, miRNAs have promising diagnostic potential in human disease including cancer [175]. However, the use of miRNA in infectious disease as diagnostic biomarkers has progressed slowly. This may have further complicated their use in cancers with viral etiology. Despite this, v-miRNAs hold enormous potential to be used as potential diagnostic biomarkers and advance precision oncology in these cancers. The potential use of miRNAs as diagnostic biomarkers is supported by their presence in various biological fluids, associated with their stability across temperature and pH ranges [176]. MiRNAs including v-miRNAs are released into the blood in various forms, which may include macrovesicles, exosomes and apoptotic bodies, and may escape host innate immune defense. MiRNAs are tissue-specific, can be detected in the blood, and are stable in the serum and plasma [177,178,179]. Due to their stability, as well as their persistent and consistent transcription throughout the course of the disease, miRNAs have been reported to serve as potent noninvasive diagnostic biomarkers and prognosticators in cancer [180]. Although reports on v-miRNA use in clinical setting is an emerging field, cellular miRNA-based therapeutics are currently undergoing pre-clinical and clinical trials, advancing their use in precision oncology [181].

Liquid biopsy, which uses body fluids such as blood to study circulating tumor cells (ctcs), cell components such as circulating tumor DNA (ctDNA), circulating tumor RNA (ctRNA), and circulating miRNAs, can also be used to study circulating v-miRNAs’ potential use in clinical cancer research, although there is a lack of reported data in this area [182,183,184]. Liquid biopsies can be used to detect circulating v-miRNAs in cancer diagnosis. This novel technique could serve as a novel, low-cost and non-invasive diagnostic and prognostic tool, thus allowing for v-miRNAs to be used as significant diagnostic biomarkers for cancer. This could replace current diagnostic procedures such as radiology, an imaging forms an important part in cancer diagnosis is expensive and, in some cases, may be harmful, while biopsies are invasive and can expose patients to medical complications such as hemorrhages [185,186]. Current cancer biomarkers, such as CA 19-9, usually used as a secondary to radiology and biopsy, have low specificity and sensitivity [187,188]. NGS and bioinformatics technologies are key in the clinical research into v-miRNAs liquid biopsies. NGS RNA Seq can be used to identify v-miRNA signatures and provide an analysis of the v-miRNome that includes novel miRNA isoforms. A bioinformatics analysis of the v-miRNome can be used to reveal molecular pathways and the associated mechanisms involved in viral induced cancer pathogenesis. Similar to oncogenic mRNA splice variants, v-miRNA isoform signatures in cancer pathogenesis may vary and this warrants further investigation in order to decipher the mechanisms employed [189]. Compared to traditional technologies such as miRNA assays, NGS technology and bioinformatics analysis can detect structural alterations in v-miRNAs, associated signatures and molecular mechanisms, in a similar manner to its ability to detect these changes in cellular miRNAs [189,190,191,192]. Circulating v-miRNAs can offer significant opportunities in the early detection, development and prognosis of these cancers [182].

**Figure 3:**
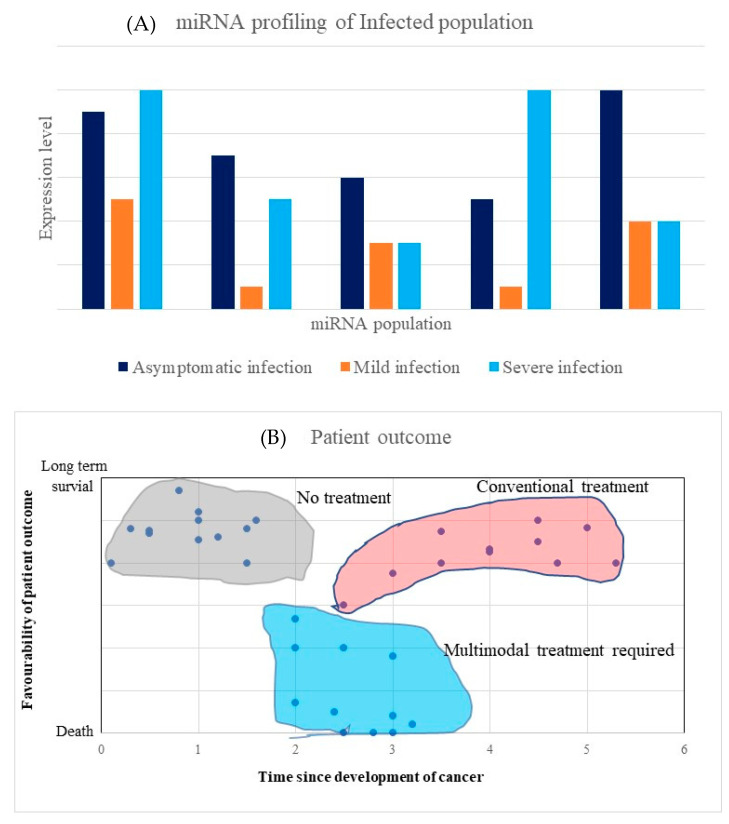
V-miRNA biomarkers in precision oncology. (**A**) v-miRNA can be profiled in an infected population. Various infections may lead to a range of disease outcomes. These may include people with no symptoms (dark blue), mild symptoms (orange) and severe symptoms (light blue). Unique v-miRNA signatures may be used to discriminate between these groups. This figure shows changes in the transcription of various hypothetical miRNAs, demonstrating how the changes in the miRNA profiles can be used to classify patients. (**B**) This will also improve cancer management and patient outcome through personalized medicine.

EBV was the first oncovirus in which v-miRNAs were discovered. Extensive work with EBV v-miRNAs in clinical research has been reported. Thus, EBV v-miRNAs can be used as a gold standard in v-miRNAs clinical applications [125]. To date, EBV antibodies are still used diagnostic markers for EBV-associated tumors [125,193]. Nonetheless, novel biomarkers for early EBV associated tumors are urgently needed. For instance, BART miRNAs (such as miR-BART 5, 7, 8, 9) have been reported to be upregulated in EBV tumor cells compared to non-tumor biopsies [194]. It has been proposed that BART miRNAs hold profound potential as novel biomarkers for EBV-associated tumors. EBV v-miRNAs have been reported to be generally upregulated compared to cellular miRNAs in NPC patients [146,195]. For example, upregulated levels of EBV-miRNAs BART2-5p and 6-5p, 17-5p were found in the serum of NPC patients compared to healthy controls [196]. Furthermore, the PTEN tumor-suppressor and the Wnt signaling pathway were shown to be targeted by these EBV v-miRNAs [197]. This study revealed a significant correlation of these BART miRNA between NPC tumor and serum samples, hence their proposed use as diagnostic and prognostic biomarkers. To complement this experimental work, computational and bioinformatics work still needs to be carried out to decipher the molecular mechanisms employed by these v-miRNAs (reviewed in [198]). Similar strategies can be used for the identification, profiling, verification, elucidation and translational application of v-miRNAs from all the oncoviruses discussed above, EBV, HPV, HBV, HCV, MCPyV and KSHV, in clinical research. Figure 3A,B illustrate the theranostic potential of v-miRNAs in clinical research and advancing precision medicine. Both illustrations use an example of the hypothetical expression of various miRNAs. In Figure 3A, various expression profiles of different v-miRNAs can be used for different prognostic or diagnostic predictions. Figure 3B shows how the expression profiles of various v-miRNAs within a population can be associated with patient outcomes and could be used to guide treatment strategies [32].

## 11. V-miRNAs’ as Novel Anti-Cancer Therapeutic Targets

Cancers with a viral etiology are a global public health problem, especially in the Sub-Saharan African (SSA) region. While other viral factors may contribute to viral oncogenesis, v-miRNAs have emerged as active role-players in viral oncogenesis [11]. There is already progress in the targeting of cellular miRNAs as anti-cancer therapeutic targets, or even as novel drugs, while targeting v-miRNA in tumorigenesis is novel. For example, a cellular miR-122 inhibitor is in a phase II clinical trial. MiR-122 is involved in HCV infection, and its inhibitor shows promising effects against infection [156,199]. It has thus been proposed that a similar antagonist mechanism can be explored against other v-miRNAs [11]. As it reduces the danger of side effects and off-targets, the anatagonist method may be more effective for v-miRNAs than for cellular miRNAs [200]. A study on murine cytomegalovirus (MCMV) in mouse models indicated that MCMV v-miRNA antagonists reduced MCMV infection in mice in vivo models [201]. Another study using anti-EBV-miRBART7-3p contained in gold nanoparticles was able to inhibit this v-miRNA due to the improved therapeutic delivery of this anti-v-miRNA, and this resulted in the inhibition of EBV-positive cells giving rise to tumors in mice [125,202]. It has also been observed that using a miRNA sponge can decrease v-miRNA activity. The EBER2 EBV promoter, for example, has been found to produce miRNA sponges, silencing target genes in EBV-infected cells [203]. In addition to their therapeutic target, v-miRNAs’ (such as EBV miR-VP-3p) use as biomarkers has also been considered [204]. Although the possibility of v-miRNAs’ use in cancer diagnosis, prognosis, and therapeutic targeting exists, much work still needs to be carried out in identifying and validating these v-miRNAs for their use in pre-clinical and clinical settings.

Like cellular miRNAs, one v-miRNA can regulate a diverse targetome. Further research is warranted into understanding host–virus miRNA gene regulatory networks. A wide-scale computational interpretation, complemented by experimental authentication in a translational oncology research setting, will be beneficial to elucidating and verifying the molecular v-miRNA carcinogenic molecular mechanisms. Due to their dual host and viral gene and network targets, multiple host regulatory pathways, such as cell-cycle checkpoints, cell proliferation and survival, cell death, host immune evasion, and tumor-suppressor pathways, are influenced [45,205]. Like cellular miRNAs, v-miRNAs hold promising therapeutic potential.

## 12. Challenges and Opportunities of v-miRNAs Use in Theranostics and Precision Oncology

One of the tasks that must be performed for v-miRNAs to be used in clinical diagnosis would be the development and validation of technologies for sample preparation and v-miRNA detection. Although there are currently advances in the detection technologies, such as Next Generation RNA Seq, industry standards for v-miRNA detection and quantification in clinical diagnosis and precision oncology are still lacking [206,207]. This is primarily due to the need to isolate these v-miRNAs from biological fluids before detection. Due to their small size, v-miRNAs may also display unclear nucleotide differences with other molecules, leading to undesired non-specificity. Even though v-miRNAs may be stable in body fluids, their delicate nature may subject them to easy degradation by RNAses during handling and processing [208].

The cost of using v-miRNA in the clinical setting is another challenge (reviewed in [209]). RNA based technologies are already considered as next-generation therapeutics and form a significant part of the biopharmaceutical industry. It is estimated that the costs associated with a new drug on the market total almost 1 billion USD, and the new drug may take an average of 10+ years to reach the market [210]. The market value of the biopharmaceutical industry in 2017 was around 218 million USD, and in 2019 this was predicted to have grown to a market value of 1.2 billion USD by the end of 2020 [211]. Various phases of clinical trials for RNA-based drugs including miRNAs are ongoing at present [212]. In addition to the forementioned challenges, adverse toxicity and side effects have been reported as a result of these miRNA-based treatments. For instance the MRX34 (microRNA liposomal injection), whose efficacy was evaluated against melanoma, has been shown to have adverse effects [213]. The absorption, distribution, metabolism and excretion (ADME) of these miRNA-based therapeutics remains to be fully elucidated. The MiRNA-based therapeutics delivery system also raises concerns (reviewed in [214]). It has also been highlighted that, even with an optimized in vivo delivery system, the localised targeted delivery of miRNA (v-miRNA) therapeutics may be a challenge and further efforts must be made to resolve these challenges (as reviewed in [215]).

EBV miRNAs’ diagnostic value as clinical biomarkers for the detection for the EBV-associated development of various tumours has not yet been clinically applied. The main challenges for the use of EBV miRNAs in the clinical setting revolves around identifying unique or common v-miRNAs that can be used as specific biomarkers for various cancers [216]. Such challenges require large-scale clinical studies to elucidate and verify their translation and application in clinical settings. The development of standardised methods to isolate and quantify these v-miRNAs from cancer patients is also warranted. While v-miRNAs translation in clinical use may be a work in progress, combining these novel molecules’ application with already existing biomarkers may be the first step in their diagnostic value.

Despite challenges in their potential use, v-miRNAs may hold various advantages regarding their potential clinical use, and these may include early detection (critical to improved patient prognosis), pathogen identification and specificity (to reduce symptom non specificity, delayed diagnosis and misdiagnosis), their ability to determine latent infections (as latent infections are key to viral induced tumorigenesis), their use in precision oncology targeted to cancers with a viral etiology (key to improved theranostics and patient outcome, as cancers of the same origin with or without viral infection may respond differently to treatment) (reviewed in [214]). The challenges, which include cell/tissue specificity, low blood detection levels and prolonged periods before detection, with routine diagnostic approaches such as using antibody detection (reviewed in [214]), may be overcome by these novel molecules. For many diseases related to viral infections, early detection and diagnosis technologies are insufficient, as these still largely rely on symptoms and pathogen antibodies whose presentation may be delayed [217]. Novel biomarkers are urgently in need to reinforce the next generation of diagnostics, particularly to improve theranostics.

## 13. Conclusions and Perspectives

Although their precise role has yet to be established, v-miRNAs play a crucial role in the viral life cycle, including the transition from lytic to latent, contributing to the persistence of viral infection, the occurrence of mutations, immune evasion and, eventually, tumorigenesis. V-miRNAs not only target and regulate viral gene expression, but also control cellular gene expression, modulating pathways that are favorable in carcinogenesis. Due to their non-immunogenicity and ability to induce immune invisibility of infected cells, v-miRNAs are promising-targets for the development of therapies and as diagnostic tools. They may also help to decipher viral-induced carcinogenesis. Deciphering the biological roles of v-miRNAs is a work in progress, and further work needs to be carried out to identify and validate v-miRNAs. Understanding the individual and corporative functions of the v-miRNA-targeted genes and their modulated pathways forms an important part of the future research to unlock v-miRNA mediated oncogenicity and aid in decoding cancers with a viral etiology and advancing precision oncology. In immunocompromised populations, viral associated cancers have been reported to have a higher prevalence, and v-miRNAs may serve as important targets in early diagnosis, improved prognosis, and therapeutic treatments. While evidence of v-miRNAs of RNA viruses is lacking, v-miRNAs biogenesis and cellular miRNA biogenesis use similar processes. MiRNAs regulate important physiological processes such as cell cycle, cell proliferation and cell death. As regulatory molecules, v-miRNAs and cellular miRNAs may share similar seed sequences, and this can pose a major challenge to key anti-proliferative processes, especially if oncogenic v-miRNA shares similar targets with an anti-tumor cellular miRNA. Thus, the competing endogenous RNA (ceRNA) network, where other non-coding RNA molecules such as the long non-coding RNAs (lncRNAs) with similar miRNA recognition elements (MREs) may serve as decoys to shield the target mRNAs, are important in anti-proliferative mechanisms. Research into the clinical use of cellular miRNAs as biomarkers in tumorigenesis, tumor progression and targeted therapy is ongoing. It is undeniable that there are various challenges in the translational application of v-miRNAs in clinical research, but these are similar to the challenges surrounding other novel strategies in clinical research. In contrast to these challenges, opportunities also exist, and these may advance personalized medicine, particularly in developing countries, where most of these cancers with viral etiologies are highly prevalent. Understanding the role of v-miRNA beyond their role in the viral life-cycle and immune evasion may unlock many doors to targeting these small molecules in viral-induced cancers.

## Figures and Tables

**Table 1:** Summary of known viral miRNAs with roles in oncogenesis.

**1. HPV miRNAs**			
**Physiological Effect**	**Cellular Target**	**Viral miRNA**	**Ref**
Immune evasion, cell cycle regulation	SP3, XRCC4, PKNOX1, JAK2, FOXP1	HPV16-miR-H2	[34]
		HPV16-miR-H2	
		HPV16-miR-H1	
		HPV6-miR-H1	
	BCL11A, TCEA1, CHD7, ITGAM, RAG1, TEF1	HPV16-miR-H3	[9,35]
		HPV16-miR-H5	
		HPV16-miR-H6	
		HPV38-miR-H1	
		HPV35-miR-H1	
		HPV68-miR-H1	
**2. HBV miRNAs**			
**Physiological Effect**	**Cellular Target**	**Viral miRNA**	**Ref**
Proliferation	PPM1A, PTEN	HBV-mir-3	[36,37]
Pro-tumorigenic	TRIM35	HBV-mir-2	[10]
**3. MCPyV miRNAs**			
**Physiological Effect**	**Cellular Target**	**Viral miRNA**	**Ref**
Immune evasion	SP100	MCV-miR-M1-5p	[38]
Viral proliferation	RUNX1	MCV-miR-M1-3p	[10]
**4. EBV miRNAs**			
**Physiological Effect**	**Cellular Target**	**Viral miRNA**	**Ref**
Immune evasion	IPO7	ebv-BART3-3p	[39]
	LMP1	ebv-BART5-5p	[40]
	RIG-1	ebv-BART6-3p	[41]
	MICB	ebv-BART2-5p	[42]
	TAP	ebv-BART17-5p	[43]
	LMP2A	ebv-BART22	[44]
	IL-12	ebv-BART1-5p	[45]
	IL1Receptor1	ebv-BHRF1-2	[46]
	CXCL-11/I-TAC TAP2	ebv-BHRF1-3	[45,47]
	*miR-122* which regulates interferons	ebv-BART1-3p	[48]
	RNF38	ebv-BART8	[49]
Angiogenesis	PHLPP1	ebv-BART15	[50]
Anti-apoptosis	TOM22	ebv-BART16	[51]
	PRDM1/Blimp1	ebv-BHRF1-2	[52]
	Bid	ebv-BART4-5p	[53]
	PUMA	ebv-BART5-5p	[40]
	P53	ebv-BHRF1-1	[54]
	BAD	ebv-BART20-5p	[55]
Tumorigenic	Catalytic subunit of AMP-activated protein kinase (AMPKα1)	ebv-BART1-5p	[56]
	LMP1	ebv-BART16	[57]
	PRDM1/BLIMP1	ebv-BHRF1-2	[52]
	ABI2	ebv-BART13-3p	[58]
Pro-metastatic	E-Cadherin	ebv-BART9	[59]
	BTRC	ebv-BART10-3p	[60]
	NDRG1	ebv-BART22	[61]
Proliferative	RIG1	ebv-BART6-3p	[41]
	FOXP1	ebv-BART11	[62]
	MAP3K5,	ebv-BART22	[63]
Viral latency	DICER	ebv-BART6-5p	[64]
**5. KSHV miRNAs**			
**Physiological Effect**	**Cellular Target**	**Viral miRNA**	**Ref**
Proliferation and metastasis	SOCS6	kshv-miR-K12-1-5p	[65]
Anti-Apoptotic	CASP3	kshv-miR-K12-1	[66]
		kshv-miR-K12-3	
		kshv-miR-K12-4	
		kshv-mir-K3	[67]
	CASP7	kshv-mir-K3	[67]
Cell migration and invasion	RK2/CXCR2/AKT Signalling	kshv-miR-K12-3	[68]
Viral latency	Rbl2	kshv-miR-K12-5	[69]
		Kshv-miR-K12-4-5p	[70]
Immune evasion	MYD88	kshv-mir-K12-5	[71]
	IRAK1,	kshv-mir-K12-9	
	C/EBP_	Kshv-mir-K12-3	[72]
		kshv-mir-K12-7	
	MICB	kshv-miR-K12-7	[73]
	IKKε	kshv-miR-K12-11	[74]
	TWEAK	kshv-miR-K12-10a	[75]
	C/EBPβ p20 (LIP)	kshv-miR-K12-3	[72]
Tumorigenesis	TGFBR2	kshv-miR-K12-10b	[76]
	CASTOR1	Kshv-miR-K4-5p,	[77]
		Kshv-miR-K1-5p,	
Regulation of lytic induction	RTA	kshv-miR-K5	[78]
		miR-K7-5p	
		kshv-miR-K9-5p,	
		kshv-miR-K3	
		kshv-miR-K4	
Differentiation of infected cells	C/EBPβ;	kshv-miR-K12-11	[3]
	MAF	kshv-miR-K12-6	[79]

Abbreviations: Abelson interactor 2 (ABI2); Bcl2-associated agonist of cell death (BAD); B-cell lymphoma/leukemia 11A (BCL11A); BH3-interacting domain death agonist (BID); Cytosolic arginine sensor for mTORC1 subunit 1 (CASTOR1); chromodomain helicase DNA binding protein 7 (CHD7); Forkhead box protein P1 (FOXP1); Integrin alpha M (ITGAM); Importin-7 (IPO7); Janus kinase 2 (JAK2); Latent membrane protein (LMP1); Musculoaponeurotic fibrosarcoma oncogene homolog (MAF); MHC class I polypeptide-related sequence B (MICB); N-myc downstream regulated gene 1 (NDRG1); PH domain and leucine rich repeat protein phosphatase 1 (PHLPP1); PBX/Knotted 1 Homeobox 1(PKNOX1); Protein phosphatase 1A (PPM1A); PR domain zinc finger protein 1, or B lymphocyte-induced maturation protein-1 (PRDM!/HLIMP1); Phosphatase **and** tensin homolog **(PTEN)**; p53 upregulated modulator of apoptosis (PUMA); Recombination activating gene 1 (RAG1); retinoblastoma (Rb)-like protein 2 (Rbl2); retinoic acid-inducible gene I (RIG1); *RING finger protein 38* *(RNF38);* *Runt-related transcription factor 1* (RUNX1); suppressor of cytokine signalling 6 (SOCS6); SP3 transcription factor (SP3); Transcription Elongation Factor A1(TCEA1); translation elongation factor 1alpha (TEF1); Translocase of outer membrane 22 (Tom22); Tripartite Motif Containing 35(TRIM35); X-ray repair cross-complementing protein 4 (XRCC4).

## Data Availability

Not applicable.
